# Novel Calibrated Short TR Recovery (CaSTRR) Method for Brain-Blood Partition Coefficient Correction Enhances Gray-White Matter Contrast in Blood Flow Measurements in Mice

**DOI:** 10.3389/fnins.2019.00308

**Published:** 2019-04-02

**Authors:** Scott W. Thalman, David K. Powell, Ai-Ling Lin

**Affiliations:** ^1^F. Joseph Halcomb III, MD Department of Biomedical Engineering, University of Kentucky, Lexington, KY, United States; ^2^Sanders-Brown Center on Aging, University of Kentucky, Lexington, KY, United States; ^3^Magnetic Resonance Imaging and Spectroscopy Center, University of Kentucky, Lexington, KY, United States; ^4^Department of Pharmacology and Nutritional Sciences, University of Kentucky, Lexington, KY, United States; ^5^Department of Neuroscience, University of Kentucky, Lexington, KY, United States

**Keywords:** arterial spin labeling, brain-blood partition coefficient, cerebral blood flow, gray-white matter contrast, magnetic resonance imaging

## Abstract

The goal of the study was to develop a novel, rapid Calibrated Short TR Recovery (CaSTRR) method to measure the brain-blood partition coefficient (BBPC) in mice. The BBPC is necessary for quantifying cerebral blood flow (CBF) using tracer-based techniques like arterial spin labeling (ASL), but previous techniques required prohibitively long acquisition times so a constant BBPC equal to 0.9 mL/g is typically used regardless of studied species, condition, or disease. An accelerated method of BBPC correction could improve regional specificity in CBF maps particularly in white matter. Male C57Bl/6N mice (*n* = 8) were scanned at 7T using CaSTRR to measure BBPC determine regional variability. This technique employs phase-spoiled gradient echo acquisitions with varying repetition times (TRs) to estimate proton density in the brain and a blood sample. Proton density weighted images are then calibrated to a series of phantoms with known concentrations of deuterium to determine BBPC. Pseudo-continuous ASL was also acquired to quantify CBF with and without empirical BBPC correction. Using the CaSTRR technique we demonstrate that, in mice, white matter has a significantly lower BBPC (BBPC_white_ = 0.93 ± 0.05 mL/g) than cortical gray matter (BBPC_gray_ = 0.99 ± 0.04 mL/g, *p* = 0.03), and that when voxel-wise BBPC correction is performed on CBF maps the observed difference in perfusion between gray and white matter is improved by as much as 14%. Our results suggest that BBPC correction is feasible and could be particularly important in future studies of perfusion in white matter pathologies.

## Introduction

Arterial spin labeling (ASL) is a non-invasive, quantitative magnetic resonance imaging (MRI) technique used to measure cerebral blood flow (CBF) in a wide variety of human conditions. A growing number of studies are using ASL to measure perfusion in a variety of preclinical murine models including, aging (Parikh et al., 2016; Hoffman et al., 2017), Alzheimer’s disease (Abrahamson et al., 2013; Lin et al., 2013, 2015), ischemic injury (Pham et al., 2010; Struys et al., 2016; Liu et al., 2017), traumatic brain injury (Foley et al., 2013), and vascular dementia (Hattori et al., 2016). This technique is based on using magnetically labeled protons on water molecules in the blood as a tracer substance to measure perfusion. As in other tracer-based techniques, in order to accurately quantify perfusion it is necessary to determine the partition coefficient of the tracer, which is in this case the relative solubility of water in the brain tissue vs. the blood. The brain-blood partition coefficient (BBPC) is tissue-specific and varies with age, species, pathology, and particularly with brain region (Herscovitch and Raichle, 1985; Kudomi et al., 2005; Leithner et al., 2010; Hirata et al., 2011). Thus the BBPC must be measured directly, and while MRI is well suited to measure water content in the brain, the current techniques to do so have prohibitively long acquisition times (Roberts et al., 1996; Leithner et al., 2010). Because of this, it is standard practice in ASL quantification to assume a BBPC value of 0.9 mL/g based on desiccation experiments performed on *ex vivo* human brain tissue (Herscovitch and Raichle, 1985; Alsop et al., 2015). This global average value is used for all regions of the brain, all ages and pathologies, and is even adopted when performing ASL in mice (Muir et al., 2008; Lei et al., 2011;Chugh et al., 2012; Gao et al., 2014).

Previous studies have determined a wide range of BBPC values in the human brain, particularly between relatively lipophilic white matter (0.82 mL/g) and hydrophilic gray matter (0.99 mL/g) (Bothe et al., 1984; Herscovitch and Raichle, 1985; Iida et al., 1989). Yet even among gray matter regions the BBPC can vary as much as 20% (Iida et al., 1989). Measurements in non-human primates have demonstrated lower BBPC values than humans with an even greater regional variability (Kudomi et al., 2005). An MRI study of BBPC in mice reported an average BBPC of 0.89 mL/g with little regional variability among gray matter regions of interest, but no white matter BBPC values were reported (Leithner et al., 2010). Because ASL has inherently low signal-to-noise ratio and the resolution requirements of scanning mouse brains are particularly high, it is necessary that the quantification methods introduce as little error as possible. Failure to correct for intra-subject regional variability as well as inter-subject variability in BBPC may result in a loss of sensitivity to perfusion deficits when using ASL. This is especially true when studying white matter regions which have both lower perfusion and lower BBPC.

In this study, we used a calibrated short TR recovery (CaSTRR) MRI sequence to measure proton density. This protocol is similar to one used previously by Leithner et al. (2010) to measure BBPC in mice, but has been modified to greatly reduce the acquisition time. Proton density was determined for the brain tissue as well as a fresh sample of each mouse’s blood placed adjacent to the animal’s head in order to calculate BBPC. Then cerebral perfusion was measured using a pseudo-continuous ASL (pCASL) technique to compare CBF maps that were uncorrected to maps that were corrected for regional BBPC. Particular attention was given to the white matter region of interest in the corpus callosum.

## Materials and Methods

All animal experiments were performed in accordance with NIH guidelines and approved by the University of Kentucky Institutional Animal Care and Use Committee (Approval number #2014-1264). Male C57Bl/6N mice aged 12 months (*n* = 8) were acquired from the National Institute of Aging colony. MRI experiments were performed using a 7T MR scanner (Clinscan, Brüker BioSpin, Germany) at the MRI and Spectroscopy Center at the University of Kentucky. Mice were anesthetized using a 4% mixture of isoflurane with air for induction and then maintained using 1.2% isoflurane such that the respiration rate was kept within 50–80 breaths/min. Rectal temperature was also monitored continually and maintained at 37 ± 1°C using a water-heated bed.

**FIGURE 1:**
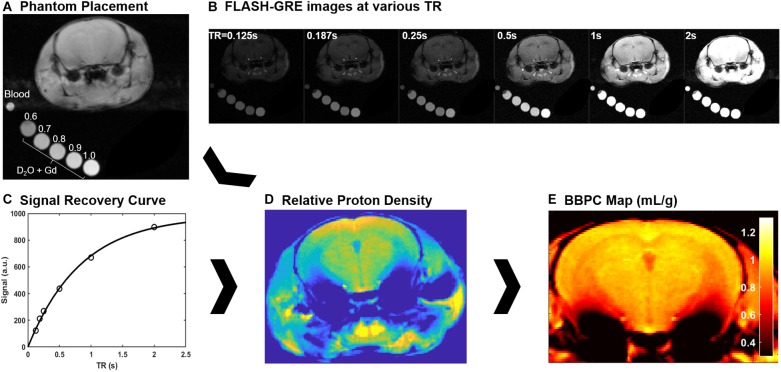
Explanation of the calibrated short repetition time recovery (CaSTRR) imaging protocol to measure BBPC. **(A)** One CaSTRR acquisition showing the placement of blood and gadolinium doped phantoms in relation to the head of the mouse. **(B)** A representative series of FLASH-GRE images used for the CaSTRR method. **(C)** A representative signal recovery curve from a single voxel of brain tissue located in the cortex region of interest (circles) along with the exponential regression used to estimate the relative proton density of the voxel (line). **(D)** A representative map of relative proton density derived from the voxel-wise signal recovery curves. **(E)** The final BBPC map calculated as the ratio of proton density in the brain to the average proton density of the blood phantom and corrected for the density of brain tissue.

While under anesthesia a fresh blood sample was taken from the facial vein and sealed in a glass capillary tube with ethylenediaminetetraactetate (EDTA) as an anticoagulant. This sample was then placed adjacent to the head of the mouse in order to measure the proton density of the blood (Figure 1A).

Both CaSTRR and pCASL images were acquired consecutively in a single imaging session. Because the CaSTRR acquisitions and the pCASL acquisitions require different receiver coils, a custom 3-D printed nose was developed to accommodate both a birdcage style volume coil and a phased-array surface coil so that the coils could be changed without disturbing the orientation of the mouse. This nose cone also facilitated the placement of phantoms adjacent to the head of the mouse.

Mice were scanned with a series of five phantoms placed alongside their head in the scanner (Figure 1A). The phantoms contained a mixture of deuterium oxide with distilled water such that the water contents of the phantoms were 60, 70, 80, 90, and 100% distilled water (Leithner et al., 2010). The phantoms were also doped with 0.07 mM gadobutrol (Gadavist, Bayer Healthcare Pharmaceuticals, Whippany NJ, United States) such that the longitudinal relaxation rate (T_1_) was similar to the T_1_ of brain tissue (∼1.6 s at 7T) (Rohrer et al., 2005).

The CaSTRR proton density measurements were acquired using a 39 mm birdcage transmit/receive coil to ensure the most uniform coil sensitivity profile possible. To measure the proton density a series of image stacks was acquired using a phase-spoiled, fast low-angle shot gradient echo (FLASH-GRE) sequence with varying repetition times (TR = 125, 187, 250, 500, 1000, 2000 ms) (Figure 1B). The shortest possible echo time (TE = 3.2 ms) was used to minimize T_2_^∗^ decay. In order to improve signal to noise, multiple averages were taken for the images with TR = 125 ms (4 averages), 187 ms (4 averages) and 250 ms (2 averages). Image matrix parameters were as follows: field of view = 2.8 cm × 2.8 cm, matrix = 256 × 256, in-plane resolution = 0.11 mm × 0.11 mm, slice thickness = 1 mm, number of slices = 10, flip angle = 90°, acquisition time = 17 min (Leithner et al., 2010).

Brain-blood partition coefficient maps were calculated in a voxel-wise manner by first fitting the signal recovery curve (Figure 1C) to the mono-exponential equation *S* = M_0_^∗^[1 – e^∧^(TR/T_1_)] to yield a map of M_0_ (Figure 1D). Next the M_0_ map was normalized to the respective phantom series by fitting a linear regression to the average M_0_ value in each phantom. Finally, the proton density in each voxel of the brain was compared to the average proton density of the blood ROI using the equation BBPC = M_0,brain_/(M_0,blood_^∗^ 1.04 g/mL) (Figure 1E; Roberts et al., 1996; Leithner et al., 2010).

For pCASL acquisitions, paired control and label images were acquired using a four-channel phased-array surface receive coil for increased signal to noise, and a whole body volume transmit coil to improve the tagging efficiency of the blood (Lin et al., 2013). Image pairs were acquired in an interleaved fashion with a train of Hanning window-shaped radiofrequency pulses of duration/spacing = 200/200 μs, flip angle = 25° and slice-selective gradient = 9 mT/m, and a labeling duration = 2100 ms. The images were acquired by 2D multi-slice spin-echo single shot echo planar imaging with FOV = 1.8 cm × 1.3 cm, matrix = 128 × 96, in-plane resolution = 0.14 mm × 0.14 mm, slice thickness = 1 mm, 6 slices, TE/TR = 20/4000 ms, label duration = 1600 ms, post-label delay = 0 s, and averages = 120. A separate, unlabeled acquisition with TR = 10 s and averages = 6 was used to normalize for the receiver coil profile. Total acquisition time for pCASL was 9 min.

When analyzing the CBF maps, the two centermost slices containing the hippocampus were selected for analysis. The brain regions of the CaSTRR and pCASL images were isolated independently using an automated skull-stripping algorithm and then co-registered using an intensity based registration algorithm. The quantitative CBF maps were calculated from the pCASL images according to the equation (Alsop et al., 2015):

$$CBF(mL/g/min)\;=\;\frac{60\;*\;BBPC\;*\;e^{\left(PLD/T_{1,blood}\right)}}{2*\alpha \;*\;(1\;-\;e^{\left(LD/T_{1,blood}\right)})}\;*\;\frac{Ctl\;-\;Lbl}{M_{0}}$$

where PLD is post-label delay, LD is label duration, T_1,blood_ is the longitudinal relaxation of blood (2.2 s at 7T), and α is label efficiency (0.85) (Alsop et al., 2015). For standard CBF maps the BBPC was assumed to be a constant 0.9 mL/g. Then a corrected CBF map was calculated by using the CaSTRR derived BBPC maps in place of the assumed constant.

Regions of interest encompassing the superior neocortex, corpus callosum, and hippocampus were drawn manually on each analyzed slice. BBPC, uncorrected CBF, and corrected CBF values were averaged for each region of interest. Gray-white contrast was determined for each slice as the absolute difference of average CBF values in gray and white matter regions of interest. All analysis was performed with in-house written scripts in Matlab (Mathworks, Natick, MA, United States).

Statistical analysis was performed using SPSS (IBM, Armonk, NY, United States). All data are expressed as mean ± standard deviation. Group comparisons were assessed using one- and two-way analysis of variance with Tukey’s *post hoc* test. Values of *p* < 0.05 were considered statistically significant.

## Results

### Corpus Callosum Demonstrates Reduced BBPC Compared to Neocortex

**FIGURE 2:**
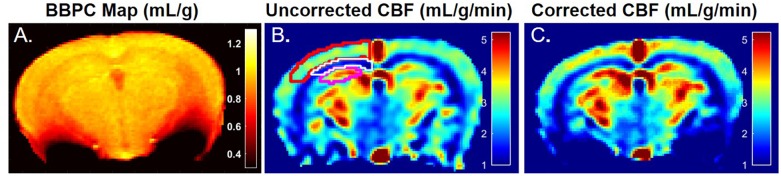
A representative map of blood-brain partition coefficient **(A)** demonstrates elevated BBPC in the neocortex relative to the corpus callosum and hippocampus (μ_Ctx_ = 0.99 ± 0.04 mL/g, μ_CC_ = 0.93 ± 0.05 mL/g, μ_Hc_ = 0.95 ± 0.04 mL/g). Maps of the uncorrected **(B)** and BBPC-corrected **(C)** cerebral blood flow (CBF) demonstrate the improved contrast between gray matter in the neocortex (top) and hippocampus (bottom) and the white matter in the corpus callosum (middle). While only one side is shown, regions of interest were drawn bilaterally and applied equally to all three maps.

**FIGURE 3:**
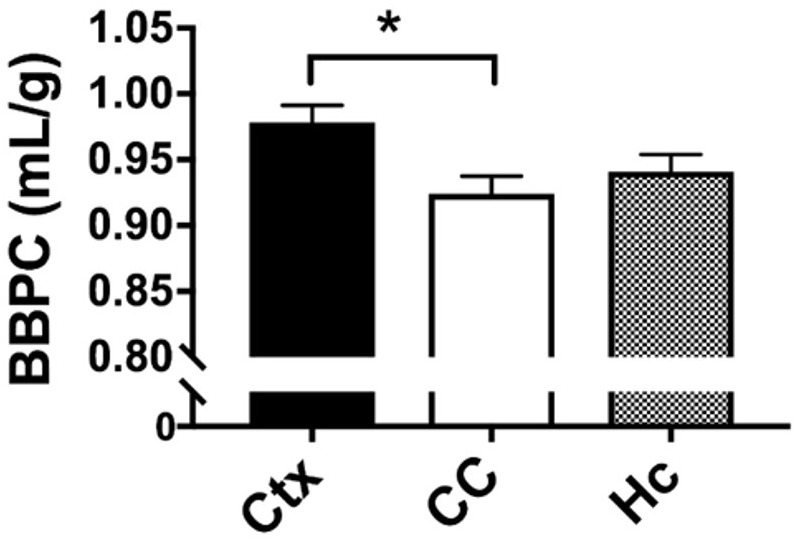
Quantitative analysis of BBPC demonstrates significantly higher BBPC in the neocortex relative to the corpus callosum (μ_Ctx_ = 0.99 ± 0.04 mL/g, μ_CC_ = 0.93 ± 0.05 mL/g, *p* = 0.035), while the hippocampus had a BBPC of μ_Hc_ = 0.95 ± 0.04 mL/g (^∗^ indicates *p* < 0.05).

The average BBPC values in the neocortex, corpus callosum, and the hippocampus were determined for each mouse and the average of all mice is reported in Table 1. The highest BBPC value was observed in the neocortex (μ_Ctx_ = 0.99 ± 0.04 mL/g) which was significantly higher than the corpus callosum (μ_CC_ = 0.93 ± 0.05 mL/g, *p* = 0.035), and also higher than the hippocampus, though not significantly (μ_HC_ = 0.95 ± 0.4 mL/g, *p* = 0.17) (Figure 2, 3).

**Table 1:** Mean partition coefficient and perfusion values by region.

Regional Values	Neocortex	Hippocampus	Corpus Callosum
BBPC (mL/g)	0.99 ± 0.04	0.95 ± 0.04	0.93 ± 0.05
CBF, uncorrected (mL/g/min)	2.81 ± 0.4	2.90 ± 0.6	1.44 ± 0.3
CBF, corrected (mL/g/min)	3.09 ± 0.5	3.07 ± 0.7	1.51 ± 0.4

**Gray-White Perfusion Contrast**	**Neocortex vs. Corpus**		**Hippocampus vs. Corpus**	

ΔCBF_Uncorrected_ (mL/g/min)	1.39 ± 0.4		1.46 ± 0.4
ΔCBF_Corrected_ (mL/g/min)	1.59 ± 0.5		1.54 ± 0.4
Contrast improvement (%, 95% CI)	14.2%, 9.6–18.8%		5.8%, 1.4–10.1%


### Corpus Callosum Also Demonstrates Lower Perfusion Than Surrounding Gray Matter

**FIGURE 4:**
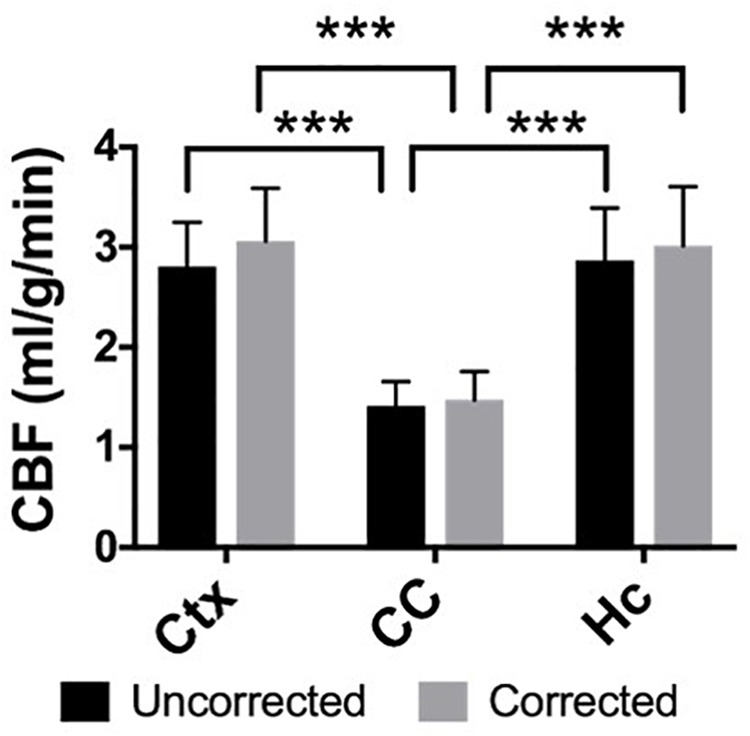
The gray matter regions of the neocortex (μ_uncorrected_ = 2.81 ± 0.4 mL/g/min, μ_corrected_ = 3.09 ± 0.5 mL/g/min) and hippocampus (μ_uncorrected_ = 2.90 ± 0.6 mL/g/min, μ_corrected_ = 3.07 ± 0.7 mL/g/min) demonstrate significantly higher CBF than the white matter corpus callosum (μ_uncorrected_ = 1.44 ± 0.3 mL/g/min, μ_corrected_ = 1.51 ± 0.4 mL/g/min) in both the uncorrected and BBPC-corrected CBF maps (^∗∗∗^ indicates *p* < 0.001).

Elevated perfusion in gray matter regions was observed relative to the corpus callosum in both uncorrected CBF maps and maps with voxel-wise BBPC correction (Figure 4). In the uncorrected maps the hippocampus demonstrated the greatest perfusion (2.90 ± 0.6 mL/g/min) followed by the neocortex (2.81 ± 0.4 mL/g/min) with significantly less perfusion in the corpus callosum (1.44 ± 0.3 mL/g/min, *p* < 0.001). However, when the maps were corrected for BBPC the perfusion in the neocortex was highest (3.09 ± 0.5 mL/g/min) followed by the hippocampus (3.07 ± 0.7 mL/g/min) with significantly less perfusion again in the corpus callosum (1.51 ± 0.4 mL/g/min, *p* < 0.001). None of the regions demonstrated significant changes in average CBF values due to BBPC correction (corrected vs. uncorrected CBF, p_Ctx_ = 0.31, p_CC_ = 0.66, p_HC_ = 0.61).

### The Difference in Perfusion Between Gray and White Matter Is Greater in Corrected CBF Maps Than Uncorrected Maps

**FIGURE 5:**
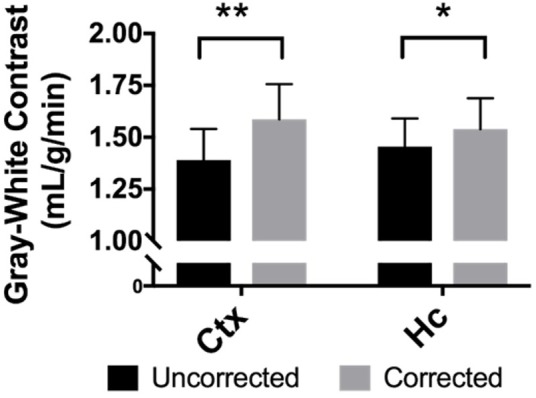
BBPC correction increased the degree of contrast between gray matter regions and the corpus callosum as measured by the absolute difference in CBF between the two regions. Contrast between the neocortex and corpus callosum was improved by 14.2% (95% CI = 9.6–18.8%, ΔCBF_uncorrected_ = 1.39 ± 0.4 mL/g/min, ΔCBF_corrected_ = 1.59 ± 0.5 mL/g/min) and between the hippocampus and corpus callosum by 5.8% (95% CI = 1.4–10.1%, ΔCBF_uncorrected_ = 1.46 ± 0.4 mL/g/min, ΔCBF_corrected_ = 1.54 ± 0.4 mL/g/min) (^∗^ indicates *p* < 0.05, ^∗∗^ indicates *p* < 0.01).

When perfusion in gray matter regions is compared to the white matter of the corpus callosum for each mouse, the average difference in perfusion for the neocortex is 1.39 ± 0.4 mL/g/min in the uncorrected maps, but it is 1.59 ± 0.5 mL/g/min in the BBPC corrected maps, this constitutes a 14.2% increase in contrast between these regions (95% CI = 9.6–18.8%). For the hippocampus the difference in perfusion is 1.46 ± 0.4 mL/g/min in the uncorrected maps and 1.54 ± 0.4 mL/g/min in the corrected maps, or a 5.8% improvement (95% CI = 1.4–10.1%) (Figure 5 and Table 1).

## Discussion

Using CaSTRR imaging we were able to produce high quality BBPC maps suitable for voxel-wise correction of perfusion measurements much faster than previous demonstrated. We determined that the average BBPC in the neocortex was 0.99 ± 0.04 mL/g and in the hippocampus the BBPC was 0.95 ± 0.4 mL/g. We also determined the BBPC in the white matter structure of the corpus callosum to be 0.93 ± 0.05 mL/g which has not previously been reported in mice. We also found significantly lower CBF in the corpus callosum than the neocortex and the hippocampus. Finally, when CBF maps were corrected for regional variability in BBPC the gray-white matter contrast was improved by as much as 14%.

The significant reduction in the acquisition time of BBPC maps to only 17 min increases the feasibility of including such a scan during an ASL protocol. We were also able to perform a voxel-wise correction due in part to the custom nose cone designed to immobilize the mouse’s head while receiver coils are changed. The result of this correction is improved sensitivity to regional perfusion differences in CBF. This study acquired high resolution BBPC maps as was done in previous studies, but those maps had to be down-sampled by 22% to match the resolution of the pCASL acquisition when calculating CBF. This means that further gains could be made in either acquisition time or signal to noise ratio by acquiring CaSTRR images at the same resolution as the ASL image. Furthermore, since the original BBPC mapping technique was adapted to use in mice from a previously established technique in humans, CaSTRR imaging should be rapidly translatable back to the clinical setting (Roberts et al., 1996). In fact, a recently published study on healthy human volunteers demonstrated that an alternative method of correcting CBF maps for BBPC variability also resulted in increased contrast between gray and white matter (Ahlgren et al., 2018). This is consistent with our study and highlights the potential benefit of BBPC correction.

The improved regional specificity of CBF maps that are corrected for BBPC variability will be particularly relevant in the study of white matter pathologies (Mutsaerts et al., 2014). There is growing interest in vascular dysfunctions that accompany commonly observed white matter pathologies like multiple sclerosis (Bester et al., 2015; Sowa et al., 2015), white matter hyperintensities (van Dalen et al., 2016), and schizophrenia (Wright et al., 2014). The inherently low signal to noise of ASL is exacerbated in white matter where there is far less perfusion than gray matter. This means differences in perfusion will be even more subtle and could be confounded by changes in BBPC. While adding a second measurement to the CBF calculation with its inherent noise may introduce more variability in the CBF maps, the ability to account for significant differences in BBPC may increase sensitivity when comparing groups or regions with small perfusion differences.

It should be noted that the CaSTRR technique differs from the one described by Leithner et al. (2010) in a few key aspects. The primary difference is the choice to use logarithmically spaced TRs and omit TRs longer than 2 s. This change reduced the acquisition time by 87% from ∼130 to 17 min. In previously published BBPC results, phantoms consisted of pure H_2_O/D_2_O solutions with very long T_1_ recovery times which necessitated long TRs (Roberts et al., 1996; Leithner et al., 2010). By adding gadolinium to the water phantoms we were able to reduce the T_1_ of the phantoms to approximately match the tissue thereby obviating the long TR scans that accounted for the vast majority of scan time. It should also be noted that Leithner et al. used 8–16 week old 129S6/SvEv mice. We would expect younger mice to have a higher BBPC than the 12 month-old mice used in our experiment, however, we observed higher BBPC values in our C57Bl/6N mice than were reported by Leithner et al. (2010). Future studies will need to consider the possibility that BBPC could vary with genetic strain.

There are several limitations to this study. While previous studies have used a uniform phantom to try and correct for the field inhomogeneity, variations were typically less than 5% and it is unlikely that the B1 field will be the same in a uniform phantom as it is while scanning a mouse (Roberts et al., 1996; Leithner et al., 2010). For this reason we chose not to perform any *post hoc* field correction and instead assumed a uniform field and receiver profile. More advanced field correction techniques may be useful. Also this study did not include a comparison to a post-mortem desiccation experiment. The standard BBPC mapping technique has been shown to underestimate the BBPC when compared to desiccation because a small fraction of water in the brain tissue does not contribute to the MRI signal (Leithner et al., 2010). Thus the overestimation of BBPC by CaSTRR may compensate for this effect, though not because it is more sensitive to this hidden water. Furthermore, regional analysis is not possible with desiccation, so desiccation could not confirm the regional differences observed by CaSTRR imaging. Finally the gradient echo readout used to acquire CaSTRR images is sensitive to susceptibility artifacts at air-tissue interfaces. This can be seen as a signal loss adjacent to the ear canals, and in this study we were forced to examine only those superior regions of the brain that were not affected by this artifact. For studies involving deep brain structures it may be necessary to separately acquire a B1 field map to correct for susceptibility variation.

In conclusion, the CaSTRR method produced maps of BBPC in mice with quality comparable to the current standard method while requiring far less acquisition time. This enables voxel-wise, empirical correction of CBF maps for regional and inter-subject variability in BBPC. These corrected CBF maps demonstrate improved contrast between gray and white matter regions. With growing interest in using ASL to measure white matter perfusion, this technique may have considerable value in studying pre-clinical models of white matter pathologies as well as potential for rapid translation to use in human studies.

## Author Contributions

ST was responsible for experimental and scanning protocol design, analysis software development, image acquisition, data and statistical analyses, and manuscript preparations. DP contributed to sequence development, scanning protocol design, technical support, and manuscript editing. A-LL was the primary investigator and contributed to project design, interpretation of results, and manuscript preparation.

## Conflict of Interest Statement

The authors declare that the research was conducted in the absence of any commercial or financial relationships that could be construed as a potential conflict of interest.
